# Case Report: Toxigenic *Corynebacterium ulcerans* Diphtheria-Like Infection in a Horse in the United Kingdom

**DOI:** 10.3389/fvets.2021.650238

**Published:** 2021-06-01

**Authors:** Flavia Zendri, Cajsa Marie Isgren, Matthew Sinovich, Peter Richards-Rios, Katie L. Hopkins, Katherine Russell, Natalie Groves, David Litt, Norman K. Fry, Dorina Timofte

**Affiliations:** ^1^Department of Veterinary Anatomy, Physiology and Pathology, Institute of Infection, Veterinary and Ecological Sciences, University of Liverpool, Neston, United Kingdom; ^2^Department of Equine Clinical Science, Institute of Infection, Veterinary and Ecological Sciences, University of Liverpool, Neston, United Kingdom; ^3^Healthcare Associated Infections and Antimicrobial Resistance Division, National Infection Service, Public Health England, London, United Kingdom; ^4^Emerging Infections and Zoonoses section, National Infection Service, Public Health England, London, United Kingdom; ^5^Respiratory and Vaccine Preventable Bacteria Reference Unit, National Infection Service, Public Health England, London, United Kingdom; ^6^Immunisation and Countermeasures Division, National Infection Service, Public Health England, London, United Kingdom

**Keywords:** emerging zoonosis, toxigenic *Corynebacterium ulcerans*, horse, respiratory diphtheria-like illness, WGS, ST543

## Abstract

*Corynebacterium ulcerans* (*C. ulcerans*) may cause diphtheria in humans and can be carried by a wide range of animal species including dairy cows and, more recently, dogs and cats that have been increasingly involved in zoonotic trasmission. We isolated and characterized, by WGS, a toxigenic *C. ulcerans* strain from a diseased horse in the United Kingdom showing clinical signs of respiratory diphtheria comparable to those seen in people. Our results indicate a role for horses as reservoirs for zoonotic *C. ulcerans*.

## Background

Diphtheria and diphtheria-like infections are caused by three *Corynebacterium* species, namely, *Corynebacterium diphtheriae, Corynebacterium ulcerans*, and *Corynebacterium pseudotuberculosis*. They can all harbor corynephage encoding the *tox* gene responsible for the production of diphtheria toxin (DT), a potentially lethal exotoxin leading to pseudomembrane formation and to the systemic signs of disease. While *C. diphtheriae* causes classic human diphtheria and carriage is almost exclusively restricted to people, *C. ulcerans* and *C. pseudotuberculosis* are mainly veterinary organisms. Nevertheless, incidents of toxigenic and non-toxigenic *tox* bearing (NTTB) *C. ulcerans* infection, with clinical pictures in people indistinguishable from those caused by *C. diphtheriae*, have been increasingly reported (1). While the global incidence of classic human diphtheria has declined globally as a result of extensive vaccination programs, *C. ulcerans* has been established as an emergent pathogen in several countries, including France, the United Kingdom, Germany, and the United States among others (2). Consequently, human infections associated with toxigenic *C. ulcerans* have exceeded those caused by toxigenic *C. diphtheriae* in many industrialized countries, including the United Kingdom (3). *C. ulcerans* zoonotic infections were originally described following livestock exposure and consumption of unpasteurized milk, but more recently, domestic pets have been progressively implicated in zoonotic transmission of *C. ulcerans* (1, 2, 4–6). Occasionally, an epidemiological link between the toxigenic *C. ulcerans* strains isolated from pets and their respective owners has been established on molecular grounds (7–11). Companion animals carrying *C. ulcerans* are often asymptomatic but may present with clinical infection, involving upper respiratory disease, particularly cats (7, 12, 13). In this study, we characterized a toxigenic *C. ulcerans* isolate recovered from a horse showing clinical signs of upper respiratory tract infection admitted to a UK university veterinary hospital (Supplementary Figure 1).

## Methods and Materials

### Culture, Antimicrobial Susceptibility Testing and Diphtheria Toxin Detection

Routine bacteriology of nasal exudate specimens was performed on nonselective media Blood Agar (BA) supplemented with 5% sheep blood and fastidious anaerobes agar (FAA, E&O Laboratories Ltd, Bonnybridge, United Kingdom). In addition, selective media for isolation and differentiation of the most common Gram-positive organisms associated with skin and mucosal surfaces (Columbia CNA Agar and Brilliance MRSA Agar) and fungi (Sabouraud dextrose agar with chloramphenicol) (Oxoid, Basingstoke, United Kingdom) were also included. Media were incubated at 37°C for bacterial organisms and at 30°C for fungi. The identification of isolates was achieved by MALDI-TOF MS (Matrix-Assisted Laser Desorption/Ionization Time-of-Flight Mass Spectrometry; MALDI Biotyper 4.1.100 Software, Bruker Daltonics, Bremen, Germany) with a score >2.2. Initial antimicrobial susceptibility testing was performed by broth microdilution using a veterinary equine susceptibility panel (TREK Diagnostic System, West Sussex, UK) and subsequently reassessed by E-test (ETEST, bioMérieux) against a human antimicrobial panel. Results were interpreted according to CLSI (14) and EUCAST (15) criteria for the veterinary and human antimicrobial panels, respectively. Confirmation of species identification and presence of the *tox* gene and toxin expression were carried out by PCR and the modified Elek Test, respectively, as previously described (16, 17) by the National Infection Service at Public Health England (PHE) (Colindale, London, UK), which contributed to the diagnostics and public health management of this case.

### Whole Genome Sequencing

Genome sequencing, assembly, and annotation of the bacterial strain were provided by MicrobesNG, Birmingham, UK (http://www.microbesng.uk). Briefly, DNA libraries were prepared using Nextera XT Library Prep Kit (Illumina, San Diego, USA) and sequencing of the chromosomal DNA of *C. ulcerans* LIV-14050 was performed using an Illumina HiSeq with a 250 bp paired-end protocol. *De novo* assembly was performed using SPAdes version 3.7 (18) and contigs ordered against the reference genome FRC11 (19) and annotated using Prokka 1.11 (20). The genome sequence of our LIV-14050 isolate was compared against six other *C. ulcerans* sequences available on GenBank [MRi49 (CP046863), FH2016-1 (AP019663), BR-AD 22 (21), *C. ulcerans* 809 (21), FRC11 (19), and NCTC7910^T^ (LT906443)] using the Artemis Comparison Tool version 13.0.16 (22). Multi-locus sequence typing (MLST) was extracted from the assembled contigs of the LIV-14050 strain using the MLST service at www.cge.cbs.dtu.dk/services/MLST/ using the MLST scheme for *C. diphtheriae* (curated at www.pubmlst.org). A minimum spanning tree diagram was developed showing sequence types (STs) of LIV-14050, the six genomes compared, and three additional isolates belonging to same ST as LIV-14050 that have been previously reported (www.pubmlst.org/cdiphtheriae). The diagram was produced using PHYLOViZ (23).

### Nucleotide Sequence Accession Number

The *C. ulcerans* LIV-14050 whole genome sequence was deposited in GenBank under accession number CP054583.

## Case Report

A six-year-old gelded warmblood cross bay horse was referred to a UK university Hospital in October 2019, following complaint of nasal discharge of 10 days duration. Upon clinical examination, the horse had right unilateral mucopurulent nasal discharge, but the exam was otherwise unremarkable. Computerized tomography revealed no sinus or dental involvement. The nasal discharge and swelling were confined to the right ventral and middle meatus, where a subsequent endoscopy demonstrated a large diphtheritic-like membrane (Supplementary Figure 2). Nasal swabs were collected for cytological and bacteriological examinations.

**Figure 1:**
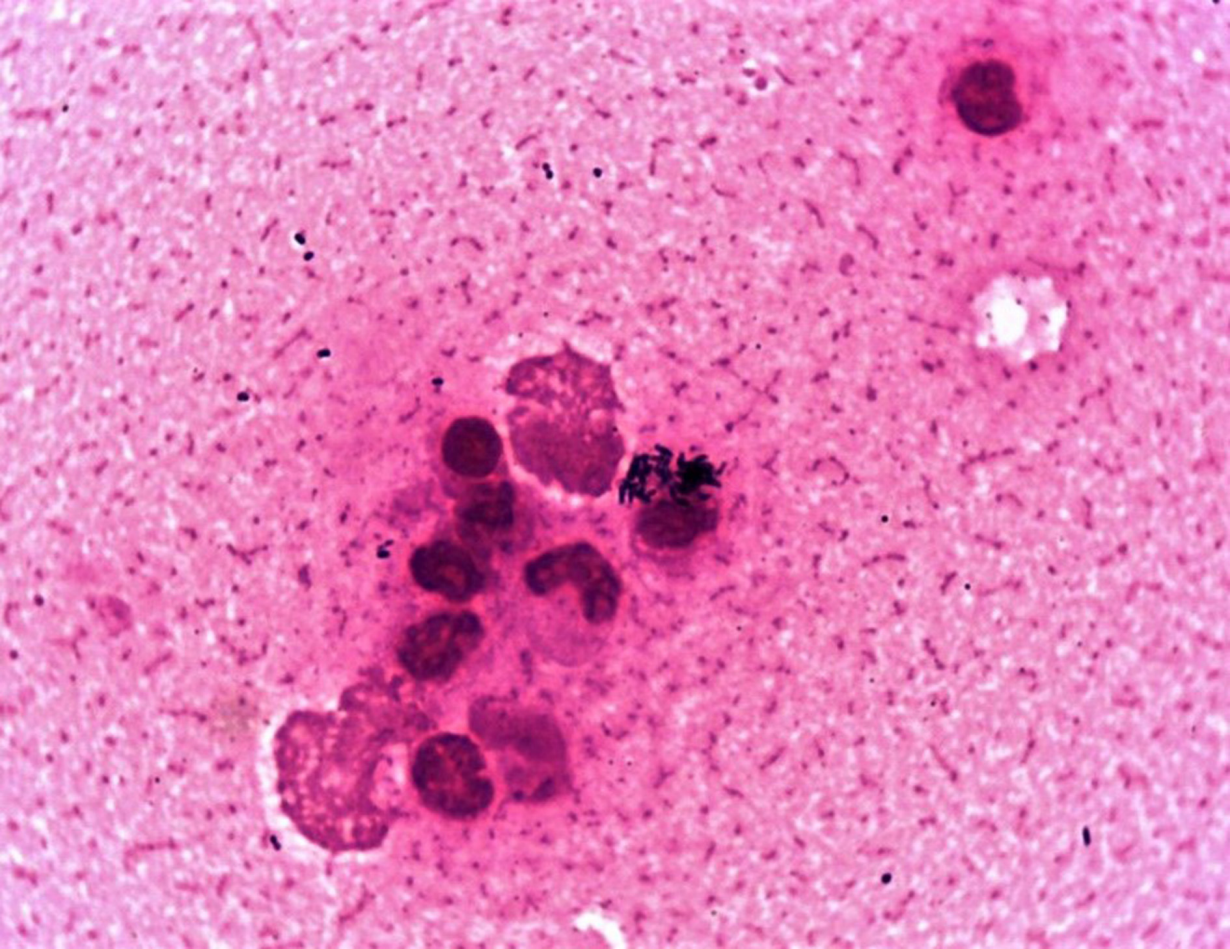
Gram-stained smear of the horse's nasal exudate showing numerous intracellular *C. ulcerans* with lesser amounts of *Streptococcus zooepidemicus*.

A bacterial culture yielded heavy mixed growths of a predominantly small, beta-hemolytic dry and waxy colony type accompanied by lesser amounts of a beta-hemolytic *Streptococcus*-like organism at 24 h post-incubation. MALDI-TOF MS identified the two organisms as *Corynebacterium ulcerans* and *Streptococcus equi* subsp. *zooepidemicus*, respectively. This was consistent with Gram-stained smears of the nasal specimen (Figure 1) and confirmed the cytological findings, which outlined the presence of active exudative rhinitis with mixed bacteria and no fungal structures. Clinicians were promptly informed of the preliminary bacteriology findings with emphasis on the zoonotic potential associated with *C. ulcerans*; as a result, the equine patient was moved to the isolation unit and infection control measures were implemented. At 48 h post-incubation, no further bacterial growth was recorded, and the *C. ulcerans* isolate, which showed more pronounced beta hemolysis, was selected for further testing. No fungal growth was recorded up to 21 days post incubation. Antimicrobial susceptibility testing results using both veterinary and human antibiotic panels are outlined in Table 1. Confirmation of bacterial species identification and presence of the *tox* gene were confirmed by PCR while toxin expression was demonstrated phenotypically by a modified Elek test. Based on the antimicrobial susceptibility results (Table 1), systemic trimethoprim-sulfamethoxazole was selected for treatment of the horse at 20 mg/kg PO every 12 h and administered with inhaled ceftiofur at 4.4 mg/kg every 24 h. Of note, our equine *C. ulcerans* isolate displayed reduced susceptibility to clindamycin (MIC = 2 mg/L), which is consistent with recent reports describing the emergence of clindamycin resistance among human and animal *C. ulcerans* isolates (2, 9, 25–27). The horse showed progressive clinical improvement until the nasal and pharyngeal lesions healed 3 weeks post-admission (Supplementary Figure 3). No *C. ulcerans* was isolated from follow-up nasal specimens obtained 4 weeks after hospital admission. At this point, all antimicrobial treatment ceased, and the patient was discharged upon full clinical recovery a week later (Supplementary Figure 1).

**Table 1:** Antimicrobial susceptibility testing of *C. ulcerans* LIV-14050 isolate by broth dilution using a veterinary panel (EQUIN2F Vet AST Plate) and by E-test (ETEST®, bioMérieux) against a human antimicrobial panel.

**Antibiotic**	**MIC (μg/ml)**	
	**Broth microdilution**	**E-test**
Penicillin	≤ 0.06 (S)	0.125 (S)
Azithromycin	≤ 0.25 (NI)	≤ 0.125 (NI)
Clarithromycin	≤ 1 (NI)	0.032 (NI)
Erythromycin	≤ 0.25 (S)	0.032 (NI)
Doxycycline	≤ 2 (S)	0.125 (NI)
Rifampicin	≤ 1 (S)	0.004 (S)
Ampicillin	≤ 0.25 (NI)	–
Ceftiofur	≤ 0.25 (NI)	–
Ceftazidime	4 (NI)	–
Amikacin	≤ 4 (NI)	–
Gentamicin	≤ 1 (S)	–
Chloramphenicol	≤ 4 (NI)	–
Enrofloxacin	≤ 0.25 (NI)	–
Imipenem	≤ 1 (S)	–
Tetracycline	≤ 2 (S)	–
Ticarcillin	≤ 8 (NI)	–
Ticarcillin-clavulanic acid	≤ 8 (NI)	–
Trimethoprim-sulfamethoxazole	≤ 0.5 (S)	–
Cefotaxime	–	1 (S)^*^
Clindamycin	–	2 (R)
Vancomycin	–	1 (S)
Linezolid	–	0.5 (S)
Ciprofloxacin	–	0.125 (S)
Moxifloxacin	–	0.064 (S)

*S, susceptible; R, resistant; NI, not interpretable*.

* *Interpretation based on the EUCAST PK-PD breakpoints (https://www.eucast.org/fileadmin/src/media/PDFs/EUCAST_files/General_documents/Organisms_and_agents_without_breakpoints_20160626.pdf)*.

**Figure 2:**
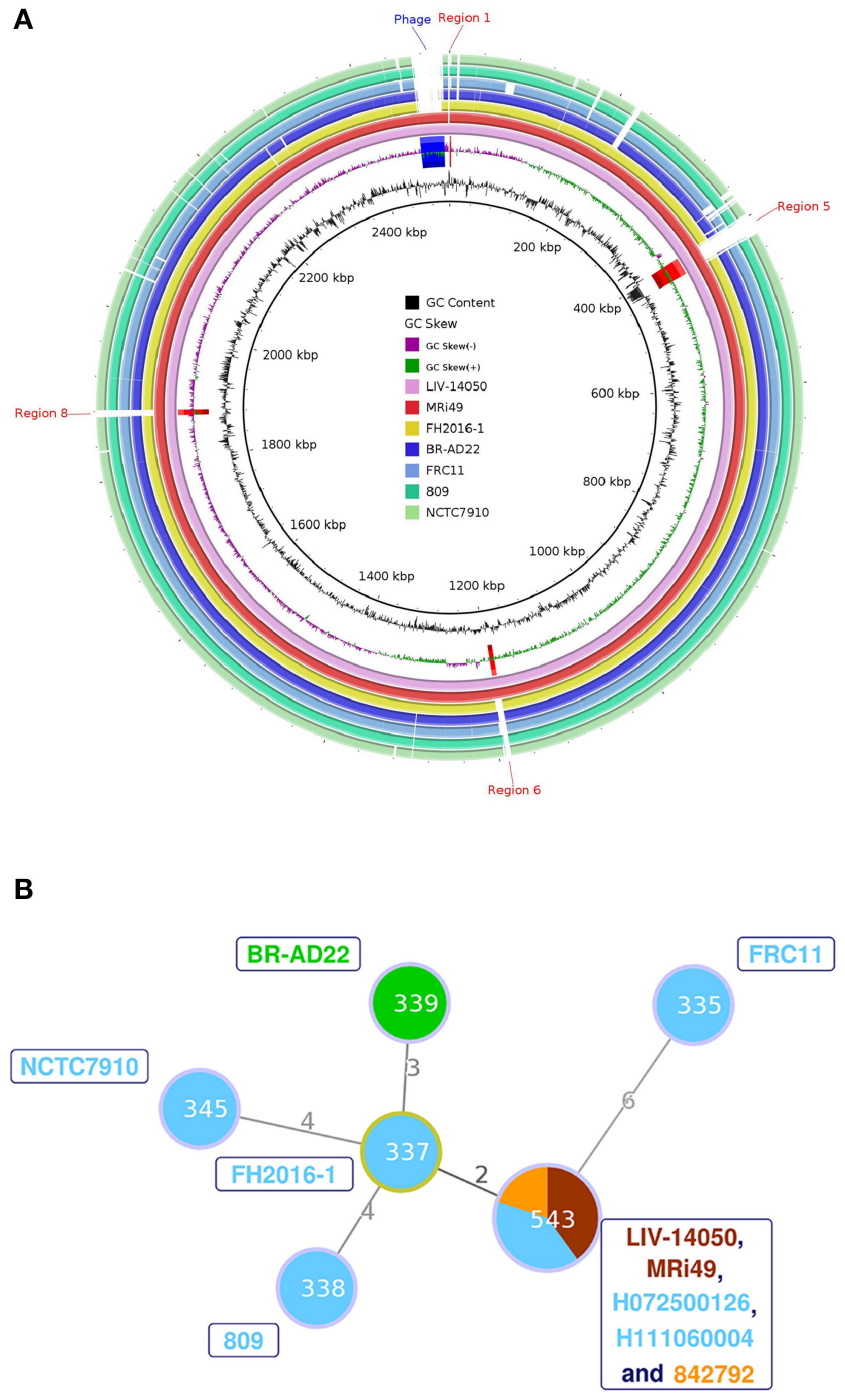
**(A)** Circular genome diagrams were produced using BLAST Ring Image Generator (BRIG) version 0.95, which uses BLAST to align the genome sequences (24). The whole genome sequence of LIV-14050 was compared against six other genomes using NCBI's online BLAST tool and visualized using the Artemis Comparison Tool (ACT) version 13.0.16 (22). Areas of the genome present in the ST453 sequences but not present in the other genomes were examined for coding sequences. A total of 24 regions and 29 genes were identified. Only the regions absent in all non-ST543 *C. ulcerans* genomes are annotated. **(B)** Minimum spanning tree diagram showing sequence types (STs) of LIV-14050, the six genomes compared in Figure 2A and the additional ST543 isolates previously reported. The size of the circle indicates the number of isolates with that ST in the data set. The colors within the circle indicate species the ST was isolated from: Human (blue); Equine (brown); Feline (orange); and Canine (green). The isolates represented by each ST are indicated by the same color labels next to the corresponding node. The numbers on the connecting edges indicate the number of nonmatching alleles between the two ST nodes.

The main genomic features of *C. ulcerans* isolate's genomic sequence, named LIV-14050, are outlined in Table 2. Of the fourteen candidate genes encoding virulence-associated proteins proposed in *C. ulcerans* (21), twelve were detected, and two, *rbp* and *vsp2*, were absent. MLST extracted from the assembled contigs identified the isolate as ST543. When compared with six published *C. ulcerans* genome sequences, all genomic features present in the other genomes were contained inside our LIV-14050 sequence. However, several genomic regions containing annotated coding sequences were found in only LIV-14050 and MRi49, a *C. ulcerans* isolate retrieved from a diseased horse in Scotland, which displayed 99.91% genomic similarity to LIV-14050 (Figure 2A). Both isolates possessed the same prophage sequence (located in LIV-14050 between 2469058-2506376 bp), shared the highest degree of similarity in virulence factor genes among the *C. ulcerans* genomes examined, and belonged to the same ST (Figure 2B), i.e., the same clonal lineage.

**Table 2:** General features of the genome sequences of *C. ulcerans* FRC11 and *C. ulcerans* LIV-14050 isolated from a human and equine patients, respectively.

**Feature**	***C. ulcerans* FRC11**	***C. ulcerans* LIV-14050**
Contigs	30	19
Genome size (bp)	2,442,826 bp	2,513,055 bp
Genome coverage	179.14x	30x
G+C content (%)	53.35%	53.21%
Total genes	2,210	2,271
Coding genes	2,146	2,183
Pseudogenes	1	30
RNA genes	65	58
Ribosomal RNAs	12	5
Transfer RNAs	51	50
Prophages	0	1
CRISPRs^*^	3 loci	3 loci

* *Clustered regularly interspaced short palindromic repeats*.

## Discussion

In the present study, we report a case of respiratory diphtheria-like infection, associated with toxigenic *C. ulcerans*, in a horse in the UK and characterized the bacterial genome sequence. To the best of our knowledge, horses represent a yet undescribed companion animal species able to carry and develop *C. ulcerans*-associated infection resembling human respiratory diphtheria. Hence, horses may be a reservoir of this zoonotic pathogen, and a differential diagnosis of respiratory diphtheria-like illness should be considered in this species based on clinical findings.

Diphtheria is a statutory notifiable disease in people in the UK, but animal-associated toxigenic *C. ulcerans* is not reportable in England. However, our preliminary microbiological findings were communicated to the national public health and veterinary authorities due to the concern over possible zoonotic implications. The equine strain was referred to Public Health England (PHE) for further diagnostics, and the local PHE team also initiated an outbreak investigation whereby comprehensive contact tracing was carried out for 49 people who had been exposed to the diseased horse. Diphtheria-containing booster vaccinations were given, and nasopharyngeal and throat swabbing was conducted on 46 people, including the horse owner, two family members, and 43 equine surgeons/veterinary students. No in-contact human tested positive for *C. ulcerans*, and the public health investigation was closed. On this occasion, the timely communication between the laboratory and the equine clinicians and their collaboration with the medical, veterinary, and public health authorities enabled the prompt confinement of the infection through adequate patient management and the implementation of human contact tracing that demonstrated the absence of *C. ulcerans* among all in-contact people.

The finding of two isolates from horses belonging to the ST543 (LIV-14050 and MRi49) in the UK raises the question whether this ST may be widely distributed in the British equine population and/or reflect a limited diversity of *C. ulcerans* in the equine population. Notably, closely related toxigenic ST543 *C. ulcerans* isolates, as shown by the minimum spanning tree diagram, have been cultured from humans with symptoms of respiratory diphtheria in the UK twice since 2007 (H072500126, H111060004), and from a cat with an infected claw (842792) [(28) and www.pubmlst.org/cdiphtheriae] indicating possible zoonotic transmission. ST543 appears to be an uncommon type; the isolates described in this paper are the only examples among 88 *C. ulcerans* isolates infecting humans (67 toxigenic, 21 nontoxigenic) and 34 veterinary isolates (27 toxigenic, 7 nontoxigenic) from between 2003 and 2020 that have been typed at PHE (pers. comm. N. Fry). Nevertheless, because of the prior detection of toxigenic *C. ulcerans* ST543 in diseased humans in the UK, further studies are warranted to explore the epidemiology and possible relationship between equine and human *C. ulcerans* infections. It would also be pertinent to determine whether the prophage is present in other ST543 isolates or observed in any other sequence types given that this genomic region appears unique to our *C. ulcerans* LIV-14050 and the MRi49 genome sequences. Furthermore, this study suggests that toxigenic *C. ulcerans* isolation and associated animal infections should be included on the list of reportable zoonotic organisms, to protect public health. This report warrants future studies on the carriage and infection of equids with *C. ulcerans* to further understand the role that infected or colonized horses may play in the epidemiology of human diphtheria-like zoonotic infections.

## Data Availability Statement

The datasets presented in this study can be found in online repositories. The names of the repository/repositories and accession numbers can be found in the article/Supplementary Material.

## Author Contributions

CI and MS led the clinical management of the equine patient. FZ and DT performed routine veterinary diagnostics. KH, DL, and NF performed further characterization of the isolate. KR and NF led the public health management of the case. PR-R, FZ, and NG analyzed and interpreted the sequencing results. FZ and DT wrote the manuscript. All authors contributed to the article and approved the submitted version.

## Conflict of Interest

The authors declare that the research was conducted in the absence of any commercial or financial relationships that could be construed as a potential conflict of interest.
