# Supplementary material for: Case Report: Toxigenic Corynebacterium ulcerans Diphtheria-Like Infection in a Horse in the United Kingdom
Source: Front Vet Sci. 2021 Jun 1;8:650238. doi: 10.3389/fvets.2021.650238 (PMC8203807; doi:10.3389/fvets.2021.650238)
Supplement: Supplementary file 1 [file Data_Sheet_1.docx]

**Supplementary Material, Figure 1.** Timeline of the clinical, laboratory, genomic and epidemiological investigations of *Corynebacterium ulcerans* equine infection (year 2019/2020).

**Supplementary Material, Figure 2.** Static endoscopic images of the right middle nasal meatus (left) and nasopharynx (right) showing diphtheritic membranes upon initial investigations following hospital admission.

**Supplementary Material, Figure 3.** Static endoscopic images of the right middle nasal meatus (left) and nasopharynx (right) showing resolution of the diphtheritic membranes three weeks after diagnosis and treatment.
