# Supplementary figures and images for: Case Report: Toxigenic Corynebacterium ulcerans Diphtheria-Like Infection in a Horse in the United Kingdom
Source: Front Vet Sci. 2021 Jun 1;8:650238. doi: 10.3389/fvets.2021.650238 (PMC8203807; doi:10.3389/fvets.2021.650238)

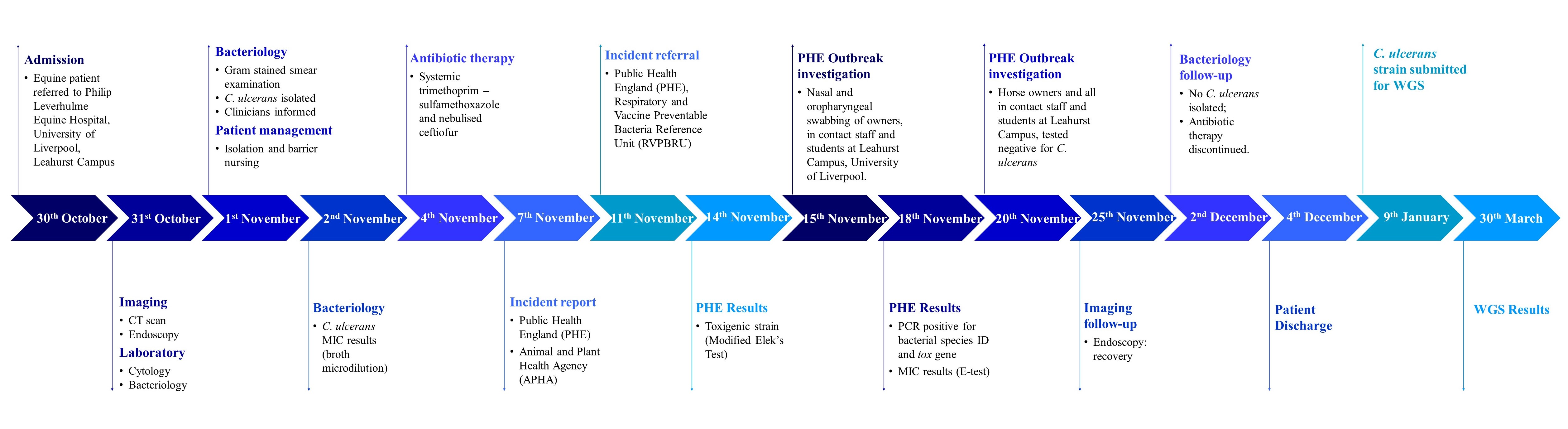

Supplement: Supplementary file 2 [file Image_1.JPEG]

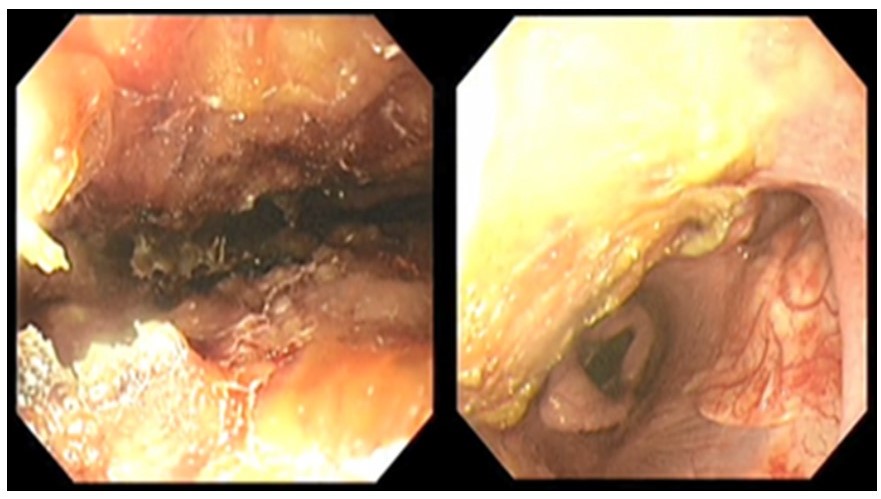

Supplement: Supplementary file 3 [file Image_2.JPEG]

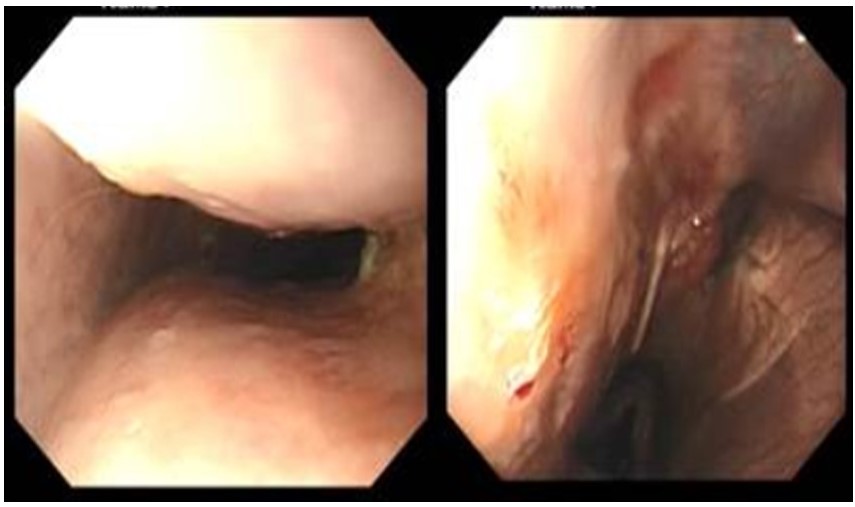

Supplement: Supplementary file 4 [file Image_3.JPEG]
